# DeepSAGE Based Differential Gene Expression Analysis under Cold and Freeze Stress in Seabuckthorn (*Hippophae rhamnoides* L.)

**DOI:** 10.1371/journal.pone.0121982

**Published:** 2015-03-24

**Authors:** Saurabh Chaudhary, Prakash C. Sharma

**Affiliations:** University School of Biotechnology, Guru Gobind Singh Indraprastha University, New Delhi, 110078, India; National Institute of Plant Genome Research (NIPGR), INDIA

## Abstract

Seabuckthorn (*Hippophae rhamnoides* L.), an important plant species of Indian Himalayas, is well known for its immense medicinal and nutritional value. The plant has the ability to sustain growth in harsh environments of extreme temperatures, drought and salinity. We employed DeepSAGE, a tag based approach, to identify differentially expressed genes under cold and freeze stress in seabuckthorn. In total 36.2 million raw tags including 13.9 million distinct tags were generated using Illumina sequencing platform for three leaf tissue libraries including control (CON), cold stress (CS) and freeze stress (FS). After discarding low quality tags, 35.5 million clean tags including 7 million distinct clean tags were obtained. In all, 11922 differentially expressed genes (DEGs) including 6539 up regulated and 5383 down regulated genes were identified in three comparative setups i.e. CON vs CS, CON vs FS and CS vs FS. Gene ontology and KEGG pathway analysis were performed to assign gene ontology term to DEGs and ascertain their biological functions. DEGs were mapped back to our existing seabuckthorn transcriptome assembly comprising of 88,297 putative unigenes leading to the identification of 428 cold and freeze stress responsive genes. Expression of randomly selected 22 DEGs was validated using qRT-PCR that further supported our DeepSAGE results. The present study provided a comprehensive view of global gene expression profile of seabuckthorn under cold and freeze stresses. The DeepSAGE data could also serve as a valuable resource for further functional genomics studies aiming selection of candidate genes for development of abiotic stress tolerant transgenic plants.

## Introduction

More than half of the crop production is adversely affected worldwide every year by various abiotic stresses such as drought, cold, heat and salinity [1]. These stresses also limit the geographical distribution of cultivated plants. Cold stress is one such environmental factor that affects plant growth and causes a significant loss to crop production [2]. Exposure to low temperature alters the expression of many genes and rehabilitates the internal structure of plant cells [3]. Plant cells also undergo various biochemical and physiological changes to cope up with chilling and freezing conditions [4]. At chilling (< 20°C) and freezing (< 0°C) temperatures, plants need to mitigate the problem of cold/freeze induced injuries in the cell membranes. Plants native to low temperature climate, on exposure to low (non freezing) temperature for a long period, show an increase in the freeze tolerance, an adaptive process called cold acclimation. Considering the importance of cold tolerance, a large number of cold responsive genes have been identified in higher plants and their regulatory mechanism has been studied at transcriptional level [5]. However, to understand the mechanism of cold stress in detail and to improve the chilling and freezing tolerance in crop plants by gene transfer, a prime and potent step is the identification and selection of genes for cold and freeze tolerance present in plants adapted to low temperature climate.

Seabuckthorn (*Hippophae rhamnoides* L.), a spiny deciduous shrub of the family Elaeagnaceae, is one of such plants that has the ability to survive in extreme environmental conditions with temperature ranging from -40°C to 40°C [6]. Since seabuckthorn is highly adaptable to varying and extreme environmental conditions, its genome is expected to harbour genes which confer tolerance to various abiotic stresses, particularly cold stress. Recently, 61 low temperature (LT) responsive extracellular proteins were reported from LT treated secretome in seabuckthorn [7], indicating the presence of a high number of LT responsive genes in seabuckthorn.

The *Hippophae* genus includes seven species, two of them probably of hybrid origin [8], native over a wide range of area covering Europe and Asia. The common seabuckthorn (*Hippophae rhamnoides* L.) is the most wide spread of all the *Hippophae* species, distributed in the cold regions of Indian Himalayas, China, Russia and many other countries of Europe and North America [9]. Seabuckthorn rapidly develops an extensive root system and, therefore, is an ideal plant for preventing soil erosion. It has a strong ability to fix atmospheric nitrogen symbiotically with *Frankia*, an actinomycetes, present in its root nodules [10]. Moreover, seabckthorn is well known for its immense nutritional and medicinal values. The pharmacological benefits of different seabuckthorn preparations include anti-tumor, anti-carcinogenic, anti-atherogenic and anti-microbial activities, immuno-modulatory and radio-protective properties [11–13]. Despite its immense ecological and medicinal value, there is scarcity of literature on molecular biology of seabuckthorn, as the main focus of research has been limited to the study of biochemical characterization and documentation of medicinal uses of seabuckthorn extracts. Nevertheless, in the last few years, seabuckthorn has attracted the attention of molecular biologists also worldwide. The limited research efforts included cloning of glycerol-3-phosphate acyltransferase (GPAT) gene and validation of its increased expression in cold-stressed leaves of seabuckthorn [14], development of EST based SSR markers [15], generation of expressed sequence tags (ESTs) [16], transcriptome study of mature seeds and fatty acid composition of seabuckthorn berries [17], *de novo* assembly of short reads of seabuckthorn transcriptome [18] and mining of microsatellites from next generation sequencing derived seabuckthorn transcriptome [19].

Gene expression profiling presents a global picture of expression of thousands of genes simultaneously by measuring the expression of mRNA in two or more experimental conditions. For global gene expression analysis, sequencing based approaches such as SAGE (Serial Analysis of Gene Expression) [20] and MPSS (Massive Parallel Signature Sequencing) [21] provide a better alternative to hybridization based microarrays [22]. Moreover, the advent of the high throughput next generation sequencing (NGS) has revolutionized the sequencing based approaches for gene expression profiling [23–26]. We employed DeepSAGE (Deep Serial Analysis of Gene Expression) [23], a combination of classical SAGE and NGS, for complete transcriptome profiling of seabuckthorn (*Hippophae rhamnoides* L.) to identify differentially expressed genes during cold and freeze stress. DeepSAGE or Deep Sequencing-based expression analysis [23, 24] provides high sensitivity, advancement in robustness and resolution, and simplifies experimental steps over other gene expression profiling techniques including classical SAGE and its modifications.

Some cold inducible elements have earlier been reported in seabuckthorn following expressed sequence tag approach [16]. However, in plants many genes are responsible for cold and freeze tolerance, therefore, a comprehensive study was required to identify cold and freeze responsive genes in seabuckthorn using a high throughput sequencing technique. In this study, we present a comprehensive view of cold and freeze responsive genes of seabuckthorn. The DeepSAGE data generated from three leaf tissue libraries (normal room temperature, cold stress at 4°C and freeze stress at -10°C), revealed a very high number of differentially expressed genes. Expression of 22 randomly selected genes was further validated by qRT-PCR (quantitative Real Time—Polymerase Chain Reaction) analysis. Our DeepSAGE data provided important details of seabuckthorn transcriptome under cold and freeze stress conditions, and insights into the understanding of cold acclimation process at molecular level. The identified sets of up regulated and down regulated genes in response to cold and freeze stress provide a valuable resource for future studies on functional genomics of abiotic stress in plants.

## Materials and Methods

### Plant Material

Seabuckthorn (*Hippophae rham*noides L.) seeds were procured from Defence Institute of High Altitude Research (DIHAR), Leh, India. Seeds were surface sterilized and germinated in dark at room temperature for 3 days. The seedlings were transferred to growth chamber, maintained at 28°C temperature, 55% relative humidity and photoperiod of sixteen hours light and eight hours dark. For differential gene expression profiling through DeepSAGE, the 30 days old plantlets, at six leaves stage, were subjected to cold stress (CS) treatment at 4°C and freeze stress (FS) at -10°C for 6 hr. The seedlings grown at 28°C were taken as control (CON). For qRT-PCR analysis, the plantlets were subjected to cold (4°C) and freeze stress (-10°C) for three time courses i.e. 2 hr, 4 hr and 6 hr.

### Total RNA Isolation

Leaf tissues were harvested from control (CON) and stress subjected plants (cold stress, CS; freeze stress, FS) and processed for RNA isolation. Total RNA from all the three samples was isolated following a modified CTAB method [27] using Intrap spin plant RNA mini kit columns (Invitek, Germany). Total RNA was quantified by gel electrophoresis and nanodrop readings (A260/A280). The RNA samples with A260/A280 ratio from 1.9 to 2.1, A260/A230 ratio from 2.0 to 2.5 and RIN (RNA Integrity Number) value of more than or equal to 8.0 were processed for further analysis.

### Tag Preparation and Illumina Sequencing

Tag preparation and sequencing was outsourced to a commercial service provider Ocimum Biosolutions, Hyderabad, India. Illumina Gene Expression Sample Prep Kit and Solexa Sequencing Chip (Flowcell) were used for tag preparation and the main instruments used for sequencing included Illumina Cluster Station and Illumina HiSeqTM 2000 System. Systematic presentation of the experimental pipeline is given in supplementary information (S1 Fig). Briefly, 6 μg of total RNA from each of the three samples (CON, CS and FS) was used for the purification of mRNA using Oligo(dT) magnetic beads. Next, Oligo(dT) primer was used to synthesize first and second strand of cDNA. The 5’ ends of the tags were generated by digestion of bead bound cDNA with restriction enzyme *Nla*III having CATG recognition site. The fragments apart from 3’ end of cDNA connected to Oligo(dT) beads were washed away and the Illumina adapter 1 was ligated to the sticky 5’ end of the digested bead bound cDNA fragments. The junction of Illumina adapter 1 and CATG site is the recognition site of *Mme*I, an endonuclease with separate recognition sites and digestion sites. It cuts at 17 bp downstream of the CATG site, producing tag with adapter 1. Illumina adapter 2 was ligated to the 3’ ends of the tags to generate a tag library by acquiring different adaptors at both ends. After 15 cycles of linear PCR amplification, 105 bp fragments were purified by 6% TBE—PAGE (Tris/Borate/EDTA—Poly Acrylamide Gel Electrophoresis). After denaturation, the single chain molecules were fixed onto the Illumina Sequencing Chip (flowcell). Each molecule grows into a single-molecule cluster-sequencing template through an *in situ* amplification process. Sequencing was performed following sequencing by synthesis (SBS) method adding four differently labeled nucleotides. Each tunnel generated millions of raw reads of 49 bp length.

### Bioinformatics Analysis of Tags

Raw sequences generated by Illumina sequencing were transformed into clean tags by removing 3’ adaptor sequences, empty reads (reads only with 3’ adaptor), low quality tags, too long and too short tags, and tags with copy number < 2. The resulting 21 nt long clean tags were further processed for alignment and annotation. The saturation analysis of sequencing was performed to confirm whether the number of detected genes increased proportionally to sequencing amount (total tag number) or not. Pearson correlation analysis was performed between parallel experimental setups viz. CON vs CS, CON vs FS, and CS vs FS to evaluate the reproducibility of DGE (Digital Gene Expression), which indicates the reliability of experimental results as well as operational stability. BLAST (version 2.2.21) and BLAST2GO (version 2.3.5) [28] analysis were performed for alignment and gene expression annotation (GO-term), respectively. All clean tags were mapped on to the reference sequences allowing only 1 bp mismatch. Tags mapped to reference sequences from multiple genes were filtered and remaining tags were designated as unambiguous clean tags. The number of unambiguous clean tags for each gene was calculated and normalized to TPM (number of transcripts per million clean tags) [24, 29].

### Screening of Differentially Expressed Genes (DEGs)

Differentially expressed genes were identified across control and stress subjected sample libraries on the basis of variation in the counts of their related sequence tags using an algorithm, a rigorous significance test, developed by Audic and Claverie [30]. The FDR (false discovery rate) ≤ 0.01 [31], and absolute value of log_2_Ratio ≥ 1 were used as threshold to judge the significance of gene expression differences. These stringent criteria and a relative threshold of 2-fold change in the sequence were used to identify differentially expressed genes in different libraries. Cluster analysis of gene expression pattern was performed using ‘cluster’ [32] and ‘Java Treeview’ [33] softwares. Pathway enrichment analysis was done for differentially expressed genes by using KEGG (Kyoto Encyclopedia of Genes and Genomes) [34], a major pathway related database.

### Validation of DEGs using qRT-PCR

Primers for the 22 randomly selected differentially expressed genes were designed using Gene Runner software version 3.05 (www.generunner.net). Total RNA was isolated from the leaf tissues of control, cold and freeze stressed plants using a modified CTAB method [27]. To remove traces of genomic DNA, the total RNA was treated with DNaseI (Fermentas). RNA quality was checked by formaldehyde gel electrophoresis and quantity measured by NanoVue (GE Healthcare). Reverse transcription reaction (25 μl) was set up using 2 μg of total RNA to construct 1^st^ strand cDNA template according to the manufacturer’s protocol (Applied Biosystem cDNA synthesis kit). Real time RT-PCR amplifications were performed in an optical 96-well PCR plate (Applied Biosystems) using One Step Plus Real Time PCR system (Applied Biosystems). First strand cDNA reaction was diluted 10 fold and 2.5 μl of it was used as a template along with Power SYBRGreen Master Mix (Applied Biosystems) and 500 nM of gene specific primer in a 25 μl Real Time PCR reaction. Universal cycling conditions (10 min 95°C, 40 cycles of 15s 95°C and 60s 60°C) were followed by generation of melting curve (obtained by heating the PCR product from 60°C to 95°C) to check the specificity of amplification. The amount of actin, a constitutive transcript (endogenous control) was normalized to check the fold change in the expression of the target genes. No template control (NTC) reaction was also included to check whether the amplification is genuine from the cDNA sample.

## Results and Discussion

### Distribution of Tags

**Fig 1:**
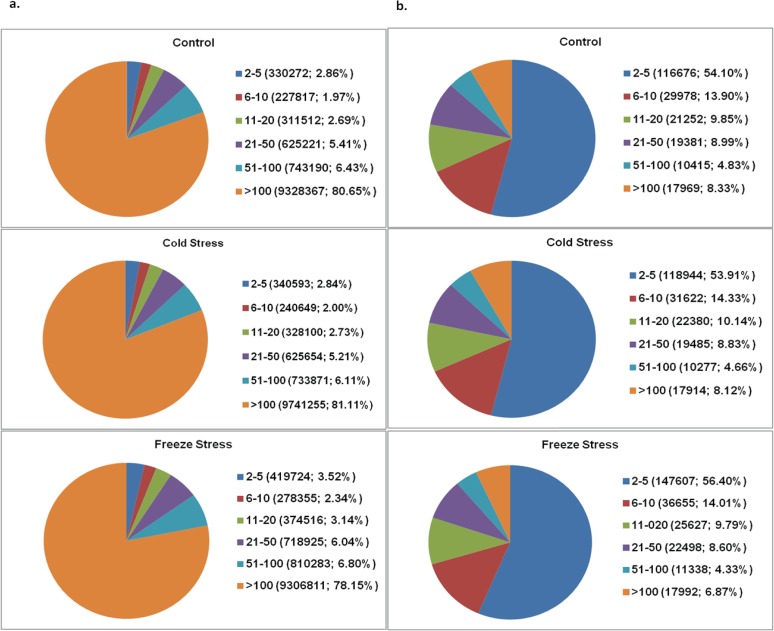
Distribution of total clean tags (a) and distinct clean tags (b), generated from digital gene expression sequencing in control, cold stress and freeze stress libraries.

DeepSAGE [23] was employed for global gene expression analysis in seabuckthorn (*H*. *rhamnoides* L.) in response to cold and freeze stress. Tags were generated using Illumina Gene Expression Sample Prep Kit and Solexa Sequencing Chip (Flowcell) from three leaf tissue cDNA libraries representing control (CON, treatment 28°C, 6 hr), cold stressed (CS, treatment 4°C, 6 hr) and freeze stressed (FS, treatment -10°C, 6 hr). Tags were further sequenced using Illumina Cluster Station and Illumina HiSeqTM 2000 System. Tag based sequencing approach, DeepSAGE, has proved to be an efficient and powerful technique for global gene expression analysis as substantial data have been generated from the three representative libraries. The sequencing depth enhanced the identification of low abundant transcripts which were beyond the reach of classical SAGE. The sequencing results showing abundance and characterization of tags from the three libraries are summarized in Table 1. In total, 36182581 raw tags including 1393681 distinct tags were generated. After filtering low quality tags including tags with copy number < 2, and too short and too long tags, 35485115 clean tags including 698010 distinct clean tags were obtained. An analysis of the distribution of tags was performed to evaluate the normality of DGE data. All three DGE libraries showed a similar pattern of distribution of total and distinct tags in different tag abundance categories. Category with smaller number of transcripts showed higher abundance whereas category with large number of transcripts exhibited very low abundance. On the basis of copy number, tags were clustered into six classes viz. 2–5, 6–10, 11–20, 21–50, 51–100 and >100 tags class. As shown in Fig 1, the proportion of tags in these six classes was found to be 55.15%, 14.11%, 9.99%, 8.66%, 4.58% and 7.6%, respectively. It was expected that tags belonging to >100 category represent lowest abundance. However, proportion of transcripts under different stresses may deviates from the expected patterns in some cases. Such a deviation has also been reported in an earlier study on cold and freeze stress [35]. This observation may reflect the high transcriptional activity of many low temperature signalling pathways in response to cold and freeze stress that are normally inactive or minimally active. Library representing freeze stress treatment had the highest number of distinct clean tags with copy number ≥ 2. This observation suggested that more number of genes are expressed during freeze stress (FS, treatment 4°C, 6 hr) as compared to control (CON, treatment 28°C, 6 hr) and cold stress (CS, treatment -10°C, 6 hr). This situation may arise due to the fact that the expression of genes changed during long exposure to stress supporting the phenomenon of cold acclimation in plants.

**Table 1:** Summary of tags generated from sequencing of Control (CON, 28°C, 6 hr), Cold Stress (CS, 4°C, 6 hr) and Freeze Stress (FS, -10°C, 6 hr) libraries.

****Summary****		****Control****	****Cold Stress****	****Freeze Stress****
Raw sequences	Total tags	11803601	12265029	12113951
	Distinct tags	452202	475010	466406
Clean tags	Total clean tags	11566379 (97.99)	12010122 (97.92)	11908614 (98.30)
	Distinct clean tags	215671 (47.69)	220622 (46.45)	261717 (56.11)
Total number of genes mapped by all clean tags	116401			
Tag mapped to sense gene	Total clean tags	3484191 (30.12)	3501960 (29.16)	3456423 (29.02)
	Distinct clean tags	60707 (28.15)	62488 (28.32)	70275 (26.85)
	Number of genes	35193 (30.23)	36491 (31.35)	39272 (33.74)
Unambiguous tag mapped to sense gene	Total clean tags	3393181 (29.34)	3408115 (28.38)	3354641 (28.17)
	Distinct clean tags	58367 (27.06)	60036 (27.21)	67467 (25.78)
	Number of genes	32722 (28.11)	33971 (29.18)	36487 (31.30)
Tag mapped to antisense gene	Total clean tags	3139369 (27.14)	3294264 (28.38)	3005305 (25.24)
	Distinct clean tags	51885 (24.06)	54011 (24.48)	59486 (22.73)
	Number of genes	30686 (26.36)	31999 (27.49)	34213 (29.39)
Unambiguous tag mapped to antisense gene	Total clean tags	3087653 (26.70)	3228056 (36.88)	2953874 (24.80)
	Distinct clean tags	50273 (23.31)	52303 (23.71)	57490 (21.97)
	Number of genes	28858 (24.79)	30105 (25.86)	32104 (27.58)
Tags mapped to gene (sense and antisense)	Total clean tags	6623560 (57.27)	6796224 (56.59)	6461728 (54.26)
	Distinct clean tags	112592 (52.21)	116499 (52.80)	129761 (49.58)
	Number of genes	43179 (37.10)	44719 (38.42)	47384 (40.7)
Unambiguous tag mapped to gene (sense and antisense)	Total clean tags	6480834 (56.03)	6636171 (55.25)	6308515 (52.97)
	Distinct clean tags	108640 (50.37)	112339 (50.92)	124957 (47.75)
	Number of genes	40440 (34.47)	41924 (36.02)	44384 (38.13)
Unknown tags	Total clean tags	4942819 (42.73)	5213898 (43.41)	5446886 (45.74)
	Distinct clean tags	103079 (47.79)	104123 (47.20)	131956(50.42)

Figures in parenthesis are in %.

### Mapping of Tags on Reference Transcriptome

All the available CATG +17 base pair clean tags were mapped on to the reference gene sequences of other species including *Vitis vinifera*, *Populus trichocarpa* and *Arabidopsis thaliana* allowing only 1 bp mismatch. Multiple genes were filtered out and remaining tags were designated as unambiguous clean tags. In total, 116401 gene reference tags were mapped with 86909 reference tags having CATG site. A total of 234300 reference tags were mapped including 225643 (96.31%) unambiguous tags. Further, 8657 (3.69%) ambiguous tags mapped to two or more genes. The number of unambiguous clean tags for each gene was calculated and then normalized to TPM (number of transcripts per million tags). Sense and antisense sequences also play an important role in the regulation of gene expression [24, 29]. Tags mapped to the complementary strand of a gene suggested that its antisense strand also has transcripts and the gene may follow the sense-antisense regulation (Table 1) in response to cold and freeze stress. The saturation analysis was also performed to check whether the number of detected genes increases with an increase in the sequencing amount (total tag number). When sequencing level reaches to 2 million or higher, the number of genes detected showed no further increase (S2 Fig). Furthermore, correlation between two parallel experiments was also analyzed for the evaluation of reliability as well as operational stability [23, 24]. The value of correlation in each analysis was very close to unity (S3 Fig) suggesting a high reproducibility of two parallel experiments (CON vs CS, CON vs FS, and CS vs FS).

### Screening of Differentially Expressed Genes (DEGs)

**Fig 2:**
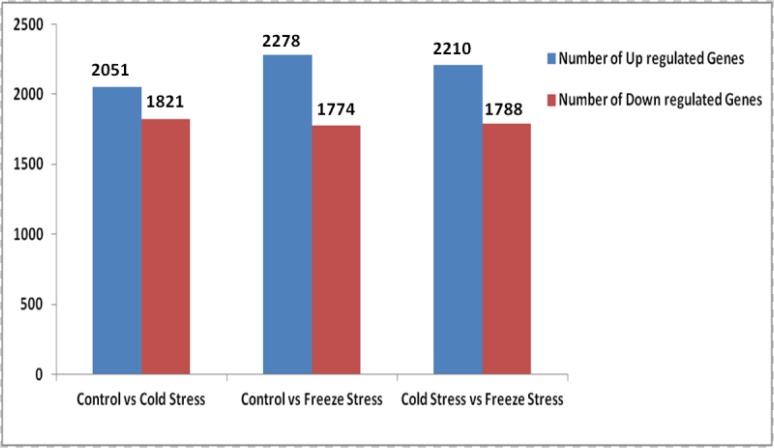
Differentially expressed genes between control (CON, treatment 28°C, 6 hr), cold stress (CS, treatment 4°C, 6 hr) and freeze stress (FS, treatment -10°C, 6 hr) libraries.

**Fig 3:**
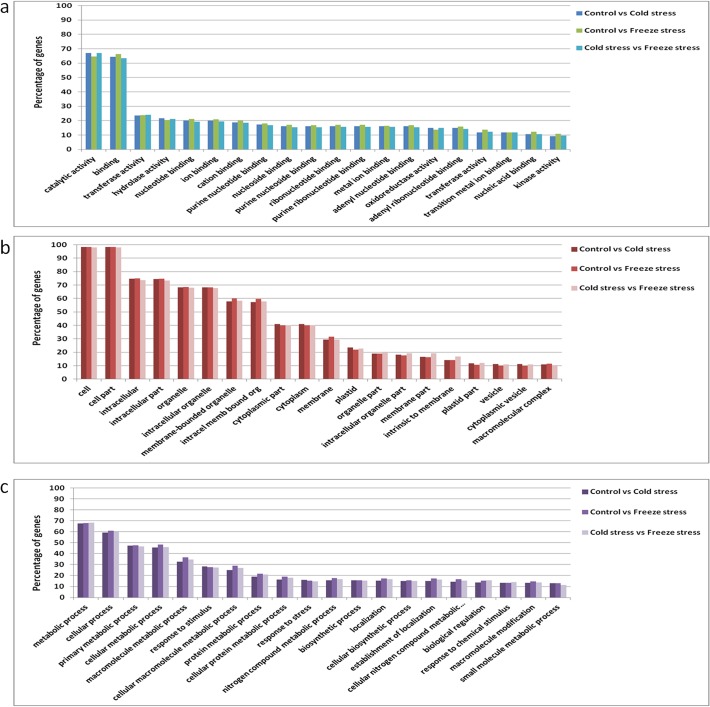
Gene Ontology terms assigned to seabuckthorn DEGs, using BLAST2GO in different categories of molecular function (a), cellular component (b) and biological process (c).

Differentially expressed genes across CON, CS and FS libraries were detected on the basis of variation in the counts of their related sequence tags using an algorithm developed by Audic and Claverie [30]. CON library was taken as control and compared with CS (cold stress) and FS (freeze stress) libraries. The latter two libraries were also compared for the identification of genes that are differentially expressed in freeze stress as compared to cold stress. The details of up regulated and down regulated genes are summarized in Fig 2. A total of 11922 genes have been identified, which are differentially expressed in the three experiments (CON, CS and FS) including 6539 up regulated and 5383 down regulated genes (S1 File). This observation supports earlier studies conducted on model plant *Arabidopsis thaliana* [36] and wheat [37], suggesting that the number of up regulated genes is higher than the number of down regulated genes under cold and freeze stress. In the present study, 53% and 56% genes were found to be cold and freeze responsive, respectively whereas 47% and 44% genes showed down regulation in response to cold and freeze stress comparative to the control. Gene ontology (GO), an international standardized gene functional classification system, was used to assign the GO-term to DEGs in CON vs CS, CON vs FS and CS vs FS sets. GO-terms were assigned to all the three categories viz. molecular function, cellular component and biological function (Fig 3). GO functional analysis was also integrated with the clustering analysis of expression patterns. In the differentially expressed genes, metabolic, cellular, primary metabolic, cellular metabolic and macromolecule metabolic processes genes were found to be abundant. Moreover, approximately 28% and 15% of the differentially expressed genes were categorized as genes responsive to stimulus and stress, respectively (Fig 3C). This observation advocates in favour of the argument that expression of large number of genes may be altered in response to external stimulus such as cold and freeze stress.

### KEGG Pathway Analysis of DEGs

Pathway enrichment analysis was performed considering the notion that different genes cooperate with each other to exercise their biological functions. KEGG pathway related database was used to identify significantly enriched metabolic and signal transduction pathways involving DEGs considering whole genome in the background. In *H*. *rhamnoides*, during cold stress, photosynthetic, carotenoid biosynthesis, and plant hormone signal transduction pathways were found to be the most significantly enriched pathways, whereas during freeze stress, photosynthetic pathway was followed by plant hormone signal transduction and vitamin B6 metabolism pathways in enrichment analysis. Therefore, our results are in agreement with the earlier studies in Arabidopsis [38], barley [39] and wheat [37], proposing that photosynthesis pathway and light play an important role in early response to cold and freezing temperatures. Further information from KEGG database indicated that both photosystems I and II (PSI and PSII) are very much influenced by cold stress. These processes transfer electrons to oxygen to form reactive oxygen species (ROS). Enrichment of carotenoid biosynthesis pathway suggested a role of carotenoids in the regulation of ABA synthesis, which might be involved in stress tolerance. Involvement of various vitamin B6 forms in abiotic stresses such as salt tolerance is well known, however, establishment of their role in cold tolerance still lacks concrete experimental evidences. In comparison to cold stress, flavonoid biosynthesis pathway was observed as most significantly enriched pathway followed by photosynthesis pathway during freeze stress. Earlier, Hannah et al. [40] have reported that genes encoding enzymes of flavonoid biosynthesis were up regulated in response to freezing tolerance in Arabidopsis. MYB transcription factors regulate the flavonoid metabolism to produce anthocyanin pigment 1 and 2 (PAP1 and PAP2). PAP1 and PAP2 transcription factors further positively associate with acclimated freezing tolerance.

The genes showing differential expression were further mapped back to our existing seabuckthorn transcriptome assembly of 88297 putative unigenes generated using next generation illumina sequencing [18]. In all, 428 genes were found to be responsive during various cold and freeze stresses in seabuckthorn (S2 File). The 22 differentially expressed genes found in seabuckthorn transcriptome were randomly selected for validation of their expression through qRT-PCR.

### Validation of Cold and Freeze Responsive Genes by qRT-PCR

**Fig 4:**
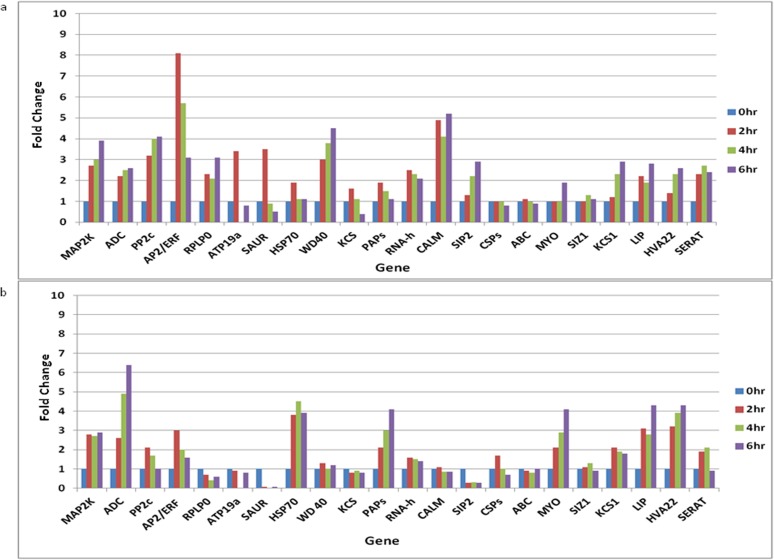
qRT-PCR validation of 22 differentially expressed genes during cold and freeze stress: a. Cold stress (4°C), b. Freeze stress (-10°C). Mitogen-activated protein kinase (*MAP2K*), arginine decarboxylase (*ADC)*, protein phosphatase 2c (*PP2c*), AP2/ERF domain-containing transcription factor (*AP2/ERF*), 60s acidic ribosomal protein p0 (*RPLP0*), peroxidase ATP 19a (*ATP19a*), SAUR family protein (*SAUR*), Heat shock protein 70 (*HSP70*), WD-40 repeat family protein (*WD40)*, Beta-ketoacyl-CoA synthase (*KCS*), plastid-lipid-associated proteins (*PAPs*), RNA helicase (*RNA-h*), calmodulin-related protein (*CALM*), galactinol—sucrose galactosyltransferase 6 (*SIP2*), cold shock protein-1 (*CSP1*), ATP binding (*ABC*), myosin (*MYO*), e3 sumo-protein ligase siz1 (*SIZ1*), 3-ketoacyl- synthase 1 (*KCS1*), low temprature induced-like protein (*LIP*), HVA 22 E protein (*HVA22*), serine acetyl transferase (*SERAT*).

To validate the differential expression of identified genes, qRT-PCR analysis was performed using total RNA isolated from the leaves of seabuckthorn plantelets subjected to cold and freeze stress for three different time-courses (2 hr, 4 hr and 6 hr). All the 22 genes used in the study with their corresponding primer sequences are listed in additional information S1 Table. Actin was used as endogenous control as its expression is reported to remain consistent under various abiotic stress conditions [41, 16]. The expression of all the genes was determined by relative quantification using 2^-ΔΔCT^ method [42]. The qRT-PCR results (Fig 4) showed conformity with the results of DeepSAGE (Table 2), suggesting DeepSAGE to be a powerful, efficient and reliable tool for global gene expression profiling in plants under various environmental stresse conditions. Furthermore, out of 22 genes selected for validation through qRT-PCR, 16 genes showed a significant up regulation and 6 genes showed down regulation in response to cold stress (Fig 4A). In freeze stress, the number of up regulated genes was found to be 12 and down regulated genes was 10 (Fig 4B).

**Table 2:** Differential expression of selected genes in response to cold (4°C, 6 hr) and freeze (-10°C, 6 hr) stress with control (28°C, 6 hr).

****Gene****	****Annotation****	****Treatment****	****Changes in Transcripts Per Million****	****Log2ratio/ Fold Change****	**Up/Down-Regulation**
*MAP2K*	Mitogen-activated protein kinase kinase	Control vs Cold	1.21 to 6.83	2.5	++
		Control vs Freeze	1.21 to 8.31	2.8	++
*ADC*	Arginine decarboxylase	Control vs Freeze	0.69 to 6.8	3.3	++
*PP2c*	Protein phosphatase 2c	Control vs Cold	0.01 to 1.42	7.1	+++
		Cold vs Freeze	79.52 to 27.54	-1.5	-
		Control vs Freeze	0.52 to 2.86	2.5	++
*AP2/ERF*	AP2 ERF domain-containing transcription factor	Control vs Cold	0.01 to 1.33	7.1	+++
*RPLP0*	60s acidic ribosomal protein p0	Control vs Cold	0.01 to 8.33	9.7	++++
		Control vs Freeze	8.33 to 0.01	-9.7	——
		Control vs Freeze	85.85 to 188.77	1.1	+
*ATP19a*	Peroxidase ATP 19a	Controlvs Cold	2.16 to 0.01	-7.8	——
		Control vs Freeze	4.76 to 1.01	-2.2	-
		Cold vs Freeze	3.75 to 1.09	-1.8	-
*SAUR*	SAUR family protein	Control vs Cold	4.93 to 0.92	-2.4	-
		Control vs Freeze	4.93 to 0.76	-2.7	—
*HSP70*	Heat shock protein 70	Cold vs Freeze	0.01 to 1.68	7.4	+++
		Control vs Freeze	0.01 to 1.68	7.4	+++
*WD40*	WD-40 repeat family protein	Control vs Cold	0.01 to 1.5	7.2	+++
*KCS*	Beta-ketoacyl-CoA synthase	Control vs Cold	4.15 to 0.5	-3.1	—-
		Control vs Freeze	4.15 to 0.25	-4.1	—-
*PAP*	Plastid-lipid-associated protein	Control vs Freeze	0.01 to 1.6	7.3	+++
*RNA-h*	RNA helicase	Control vs Cold	0.01 to 1.58	7.3	+++
*CALM*	Calmodulin-related protein	Control vs Cold	0.01 to 3.41	8.4	++++
		Cold vs Freeze	26.98 to 6.3	-2.1	-
*SIP2*	Galactinol—sucrose galactosyltransferase 6	Control vs Cold	4.93 to 17.98	1.9	+
		Cold vs Freeze	17.98 to 2.35	-3	—
*CSPs*	Cold shock protein-1	Control vs Cold	2.07 to 0.25	-3	—
		Cold vs Freeze	8.16 to3.86	-1.1	-
		Control vs Freeze	3.11 to 0.01	-8.3	——
*ABC*	ATP binding	Control vs Cold	1.73 to 0.01	-7.4	—-
*MYO*	Myosin	Control vs Freeze	0.01 to 2.02	-7.7	——
*SIZ1*	e3 sumo-protein ligase siz1	Control vs Freeze	1.21 to 0.01	-6.9	—-
*KCS1*	3-ketoacyl- synthase 1	Control vs Cold	1.04 to 16.57	4	++
		Control vs Freeze	1.04 to 5.88	2.5	++
*LIP*	Low temprature induced-like protein	Control vs Freeze	0.01 to 1.6	7.3	+++
		Control vs Freeze	4.58 to 11.25	1.3	+
*HVA22*	HVA 22 E protein	Control vs Freeze	0.01 to1.93	7.5	++++
		Control vs Freeze	0.86 to 4.45	2.3	+
*SERAT*	Serine acetyl transferase	Control vs Cold	0.01 to 1.25	7	+++
		Cold vs Freeze	45.13 to 2.27	-4.3	—

“+” sign indicates the intensity of up regulation whereas “-” indicates the intensity of down regulation of differentially expressed genes.

### Expression of Cold and Freeze Responsive Genes

According to the study by Thomashow [5] on plant cold acclimation, many cold responsive genes in plants encode various signal transduction and regulatory proteins such as mitogen activated protein kinase kinase (MAPK kinase) and calmodulin-related proteins. These proteins enhance the freezing tolerance by controlling the expression of genes and regulating the activity of proteins involved in freezing tolerance in plants. Also, genes encoding chaperons like hsp70 contribute to freezing tolerance by stabilizing proteins against freeze induced denaturation. In *H*. *rhamnoides*, genes encoding MAPK kinase, calmodulin-related proteins and heat shock proteins were differentially expressed when plants were subjected to cold and freeze stress. Genes encoding signal transduction proteins (MAPK kinases) and regulatory proteins (calmodulin-related proteins) showed more than two fold increase in their expression during early response to cold stress. Furthermore, the MAPK kinase activity showed up regulation in response to freeze stress as the time period progresses whereas expression of genes for calmodulin related proteins got inhibited during freeze stress. The heat shock protein (hsp70) gene expression showed a mild increase in response to cold stress comparative to a 3–4 fold increase during freeze stress suggesting its role in mediating freezing tolerance in seabuckthorn.

In the present study, there was a marked increase in the expression of genes encoding various enzymes such as arginine decarboxylase, protein phosphates, serine acetyl transferase, ketoacyl synthase 1 and RNA helicase under cold as well as freeze stress. These enzymes play significant roles in the biosynthesis and metabolism of important compounds, which further regulate the cold and freeze tolerance in plants. For example, arginine decarboxylase (ADC) participates in the biosynthesis of polyamines that increase the abiotic stress tolerance especially cold tolerance in plants [43]. In *H*. *rhamnoides*, gene encoding ADC showed significant change of 2–3 fold increase in cold stress concurrent with a 6–7 fold change under freeze stress suggesting a pivotal role of polyamines metabolism in freeze stress tolerance. Also, protein phosphatase, ketoacyl synthase 1 and RNA helicase mediate cold and freeze tolerance in plants by negatively regulating ABA synthesis, metabolizing long chain fatty acids and regulating transcription factors [44–47]. However, gene encoding other forms of ketoacyl synthase i.e. beta-ketoacyl synthase showed down regulation in freezing conditions as well as on long exposure to cold stress, indicating variable roles of different members of the same gene family during abiotic stress. The other genes encoding enzymes such as peroxidase, galactinol-sucrose galactosyltransferase and sumo-protein ligase showed mild increase in the expression or down regulation in cold and freeze stress (Fig 4).

The role of AP2/ERF domain-containing transcription factors and their involvement in cold inducible gene expression is well understood in Arabidopsis [47]. The genes encoding these types of transcription factors were also found up regulated by 8 fold in early response to cold stress and 3 fold in response to freeze stress in our study. In Arabidopsis, WD40-repeat family proteins that repress expression of genes associated with abiotic stress tolerance through histone deacetylation have been identified and characterized [48]. Mutation in gene encoding HOS15, a member of WD-repeat family protein, provides acclimation and tolerance to cold stress in Arabidopsis through chromatin remodelling. In the present study, genes for WD-repeat family proteins were also found 4 fold up regulated on exposure to cold stress for 6 hr, suggesting their crucial role in cold acclimation in seabuckthorn. In response to cold stress, the genes encoding proteins such as plastid lipids associated proteins and myosin showed a marginal increase in the expression whereas genes for low temperature induced proteins and HAV 22E proteins showed more than 2 fold increase in their expression. Furthermore, these genes showed a high increase in the level of expression (2–4 folds) in response to freeze stress in our study (Fig 4). Lee et al. [49] in their study of cold responsive transcriptome of Arabidopsis observed that expression of many auxin related genes was down regulated in response to cold and majority of them belonged to SAUR family genes. It was further suggested that SAUR family gene transcripts are very unstable under cold stress, either because of reduced auxin level or decrease in their transcripts. A similar trend was observed in our study also as the genes for SAUR family proteins were down regulated on exposure of *H*. *rhamnoides* to cold and freeze temperatures. The genes encoding proteins of nucleus proteome like 60s ribosomal protein showed an up to 3 fold increase in their expression under cold stress suggesting the importance of *de novo* protein synthesis under environmental stress. However, the expression of genes encoding ribosomal protein got inhibited under freeze stress. This may reflect the disruption of signalling pathways related to cold stress on long exposure to low temperature because of dehydration as suggested by Kim et al. [50] in soybean. Furthermore, gene for ATP binding protein, a member of membrane transport proteins, showed down regulation in response to cold and freeze stress in our study (Fig 4). An important observation in our study was the down regulation of cold shock protein genes under cold and freeze stress (Fig 4). Role of cold shock domain (CSD) protein under prolonged exposure to cold is well understood and characterized during cold acclimation in winter wheat (WCSP1), rice (OsCSP1) and Arabidopsis (AtCSP1) [51]. However, the genes for cold shock protein (CSP1) in seabuckthorn showed down regulation in response to cold and freeze stress. This observation suggested the need of further investigation of biological and cellular functions of cold shock domain proteins in plants including seabuckthorn.

## Conclusions

Seabuckthorn (*H*. *rhamnoides* L.) genome is expected to harbour genes which might confer resistance to various abiotic stresses specifically cold and freeze stress considering its adaptability to varying and extreme environments. DeepSAGE, a tag based profiling approach proved to be an efficient and powerful tool for the study of global gene expression in seabuckthorn providing a comprehensive view of genes differentially expressed during cold and freeze stress. DeepSAGE data of seabuckthorn provided useful resource and reference dataset for further functional genomics analysis in seabuckthorn and other important crops. The present study implicated a large number of genes with different biological functions expressing differentially in response to cold and freeze stress treatment. Isolation and further characterization of these genes will help researchers in understanding their role in cold and freeze tolerance in seabuckthorn and may provide important gene resources to be exploited for the development of stress tolerant crop plants in future.

## Supporting Information

S1 Fig Experimental pipeline of digital gene expression profiling (A), procedure of bioinformatics analysis for digital gene expression profiling (B). (PPT)

S2 Fig Sequencing saturation analysis in three libraries representing control (A), cold stress (B) and freeze stress (C). (PPT)

S3 Fig Correlation analysis between two parallel experimental setups. (A) Control vs Cold Stress (CON vs CS), (B) Control vs Freeze Stress (CON vs FS), (C) Cold Stress vs Freeze Stress (CS vs FS).
(PPT)

S1 File The differentially expressed genes between control, cold stress and freeze stress treated plants. TPM: transcript per million tags. FDR: false discovery rate. We used “FDR≤0.001 and the absolute value of log2Ratio≥1” as the threshold to judge the significance of gene expression difference.
(XLS)

S2 File List of 428 differentially expressed cold and freeze responsive genes identified from seabuckthorn transcriptome assembly. (XLS)

S1 Table List of genes and primers used in qRT-PCR analysis. (DOC)
