# Supplementary material for: DeepSAGE Based Differential Gene Expression Analysis under Cold and Freeze Stress in Seabuckthorn (Hippophae rhamnoides L.)
Source: PLoS One. 2015 Mar 24;10(3):e0121982. doi: 10.1371/journal.pone.0121982 (PMC4372589; doi:10.1371/journal.pone.0121982)
Supplement: S1 Fig — (PPT) [file pone.0121982.s001.ppt]

## Slide 1
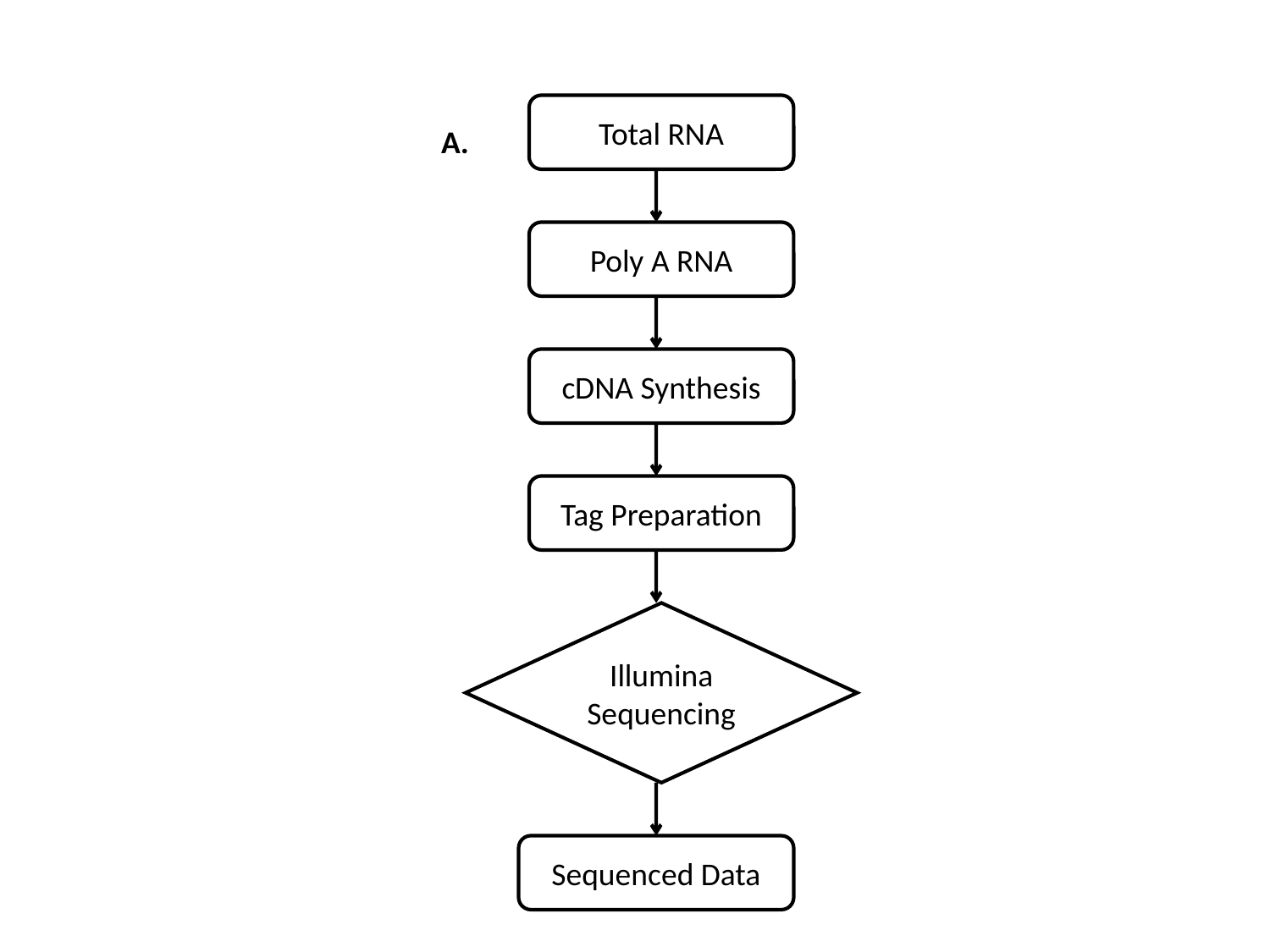

A.
Total RNA
Poly A RNA
cDNA Synthesis
Tag Preparation
Illumina Sequencing
Sequenced Data

## Slide 2
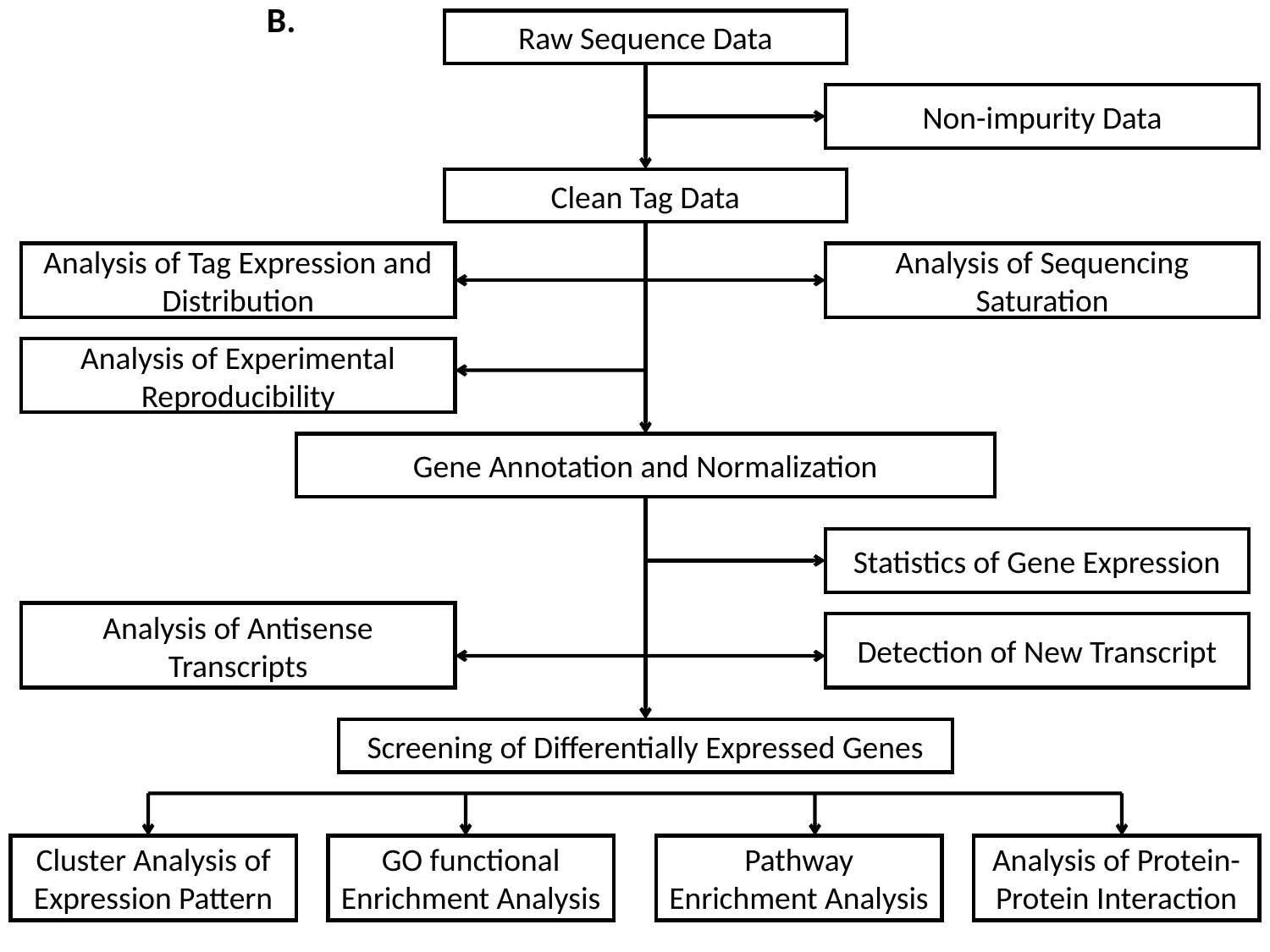

B.
Raw Sequence Data
Non-impurity Data
Clean Tag Data
Analysis of Tag Expression and Distribution
Analysis of Sequencing Saturation
Analysis of Experimental Reproducibility
Gene Annotation and Normalization
Statistics of Gene Expression
Analysis of Antisense Transcripts
Detection of New Transcript
Screening of Differentially Expressed Genes
Cluster Analysis of Expression Pattern
GO functional Enrichment Analysis
Pathway Enrichment Analysis
Analysis of Protein- Protein Interaction
