# Supplementary figures and images for: DeepSAGE Based Differential Gene Expression Analysis under Cold and Freeze Stress in Seabuckthorn (Hippophae rhamnoides L.)
Source: PLoS One. 2015 Mar 24;10(3):e0121982. doi: 10.1371/journal.pone.0121982 (PMC4372589; doi:10.1371/journal.pone.0121982)

## Slide 1
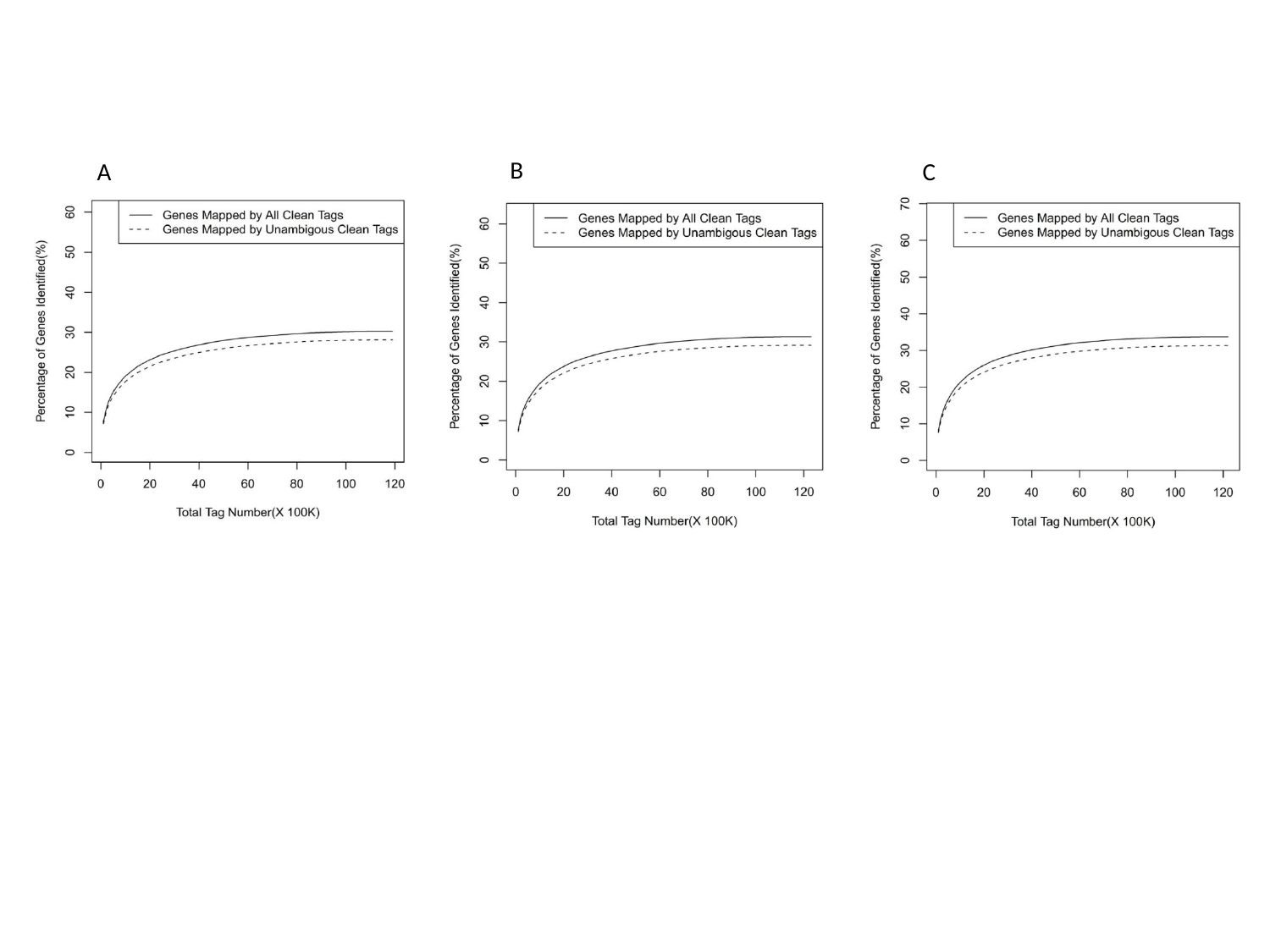

B
A
C

Supplement: S2 Fig — (PPT) [file pone.0121982.s002.ppt]

## Slide 1
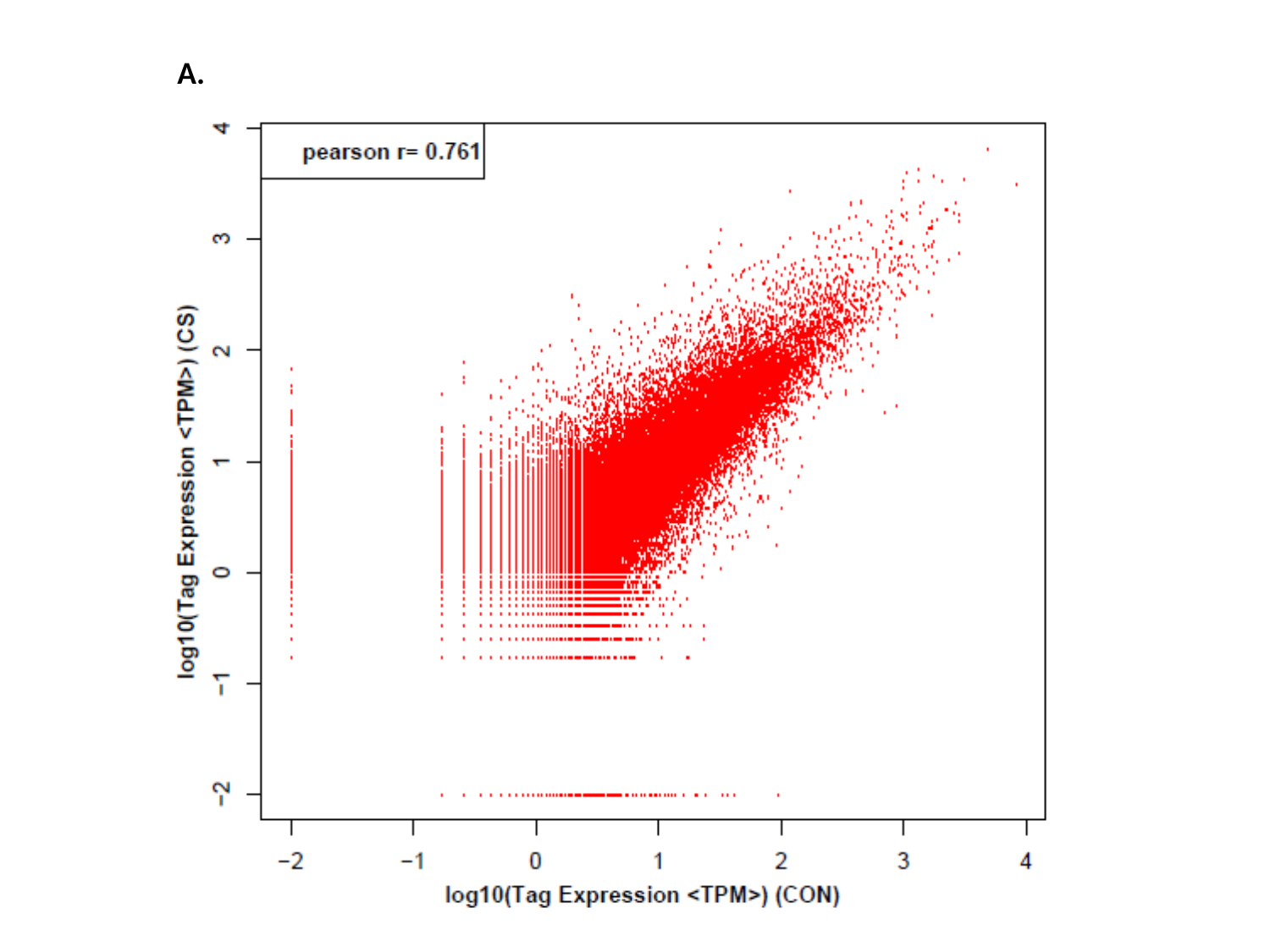

A.

## Slide 2
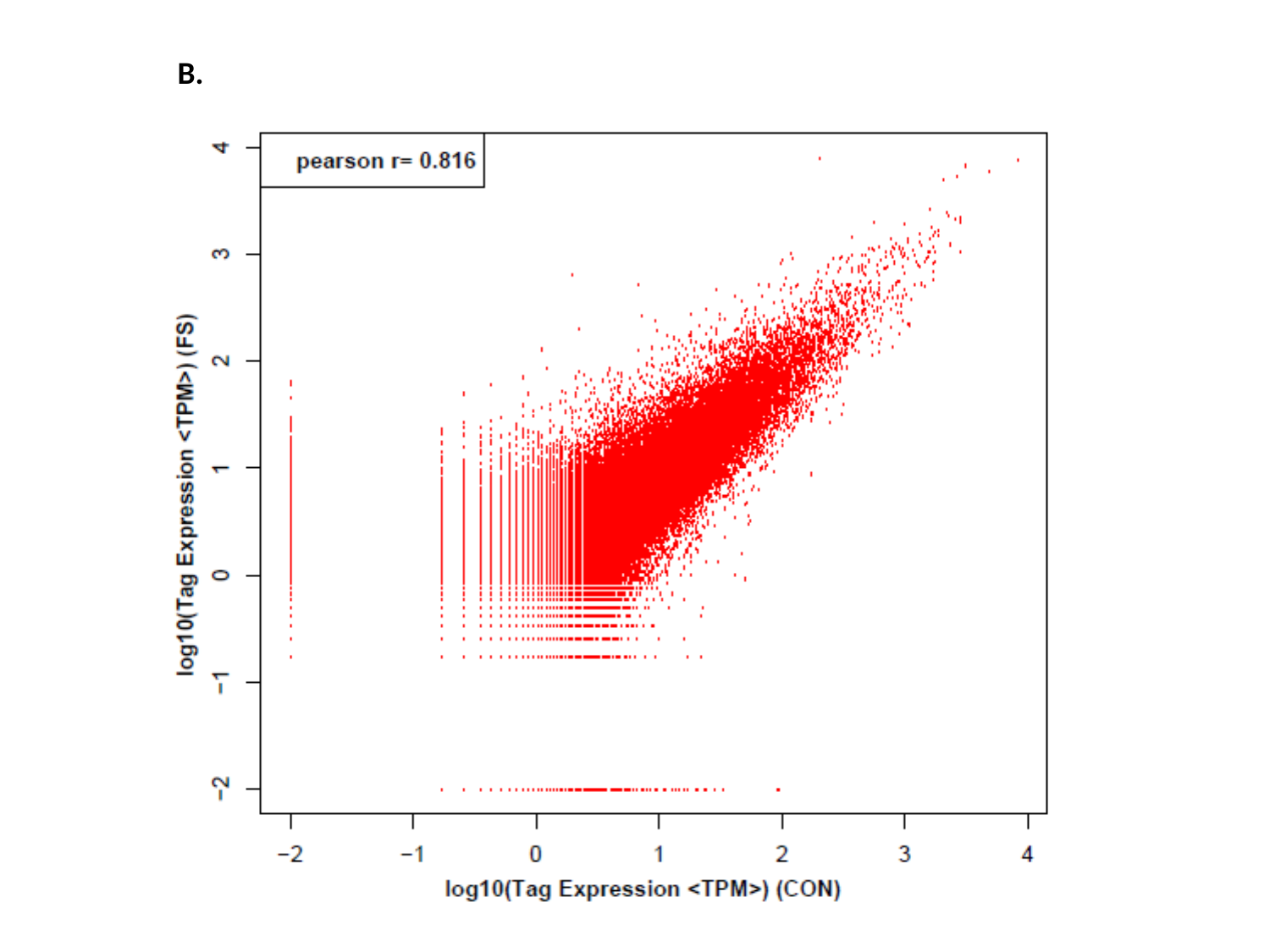

B.

## Slide 3
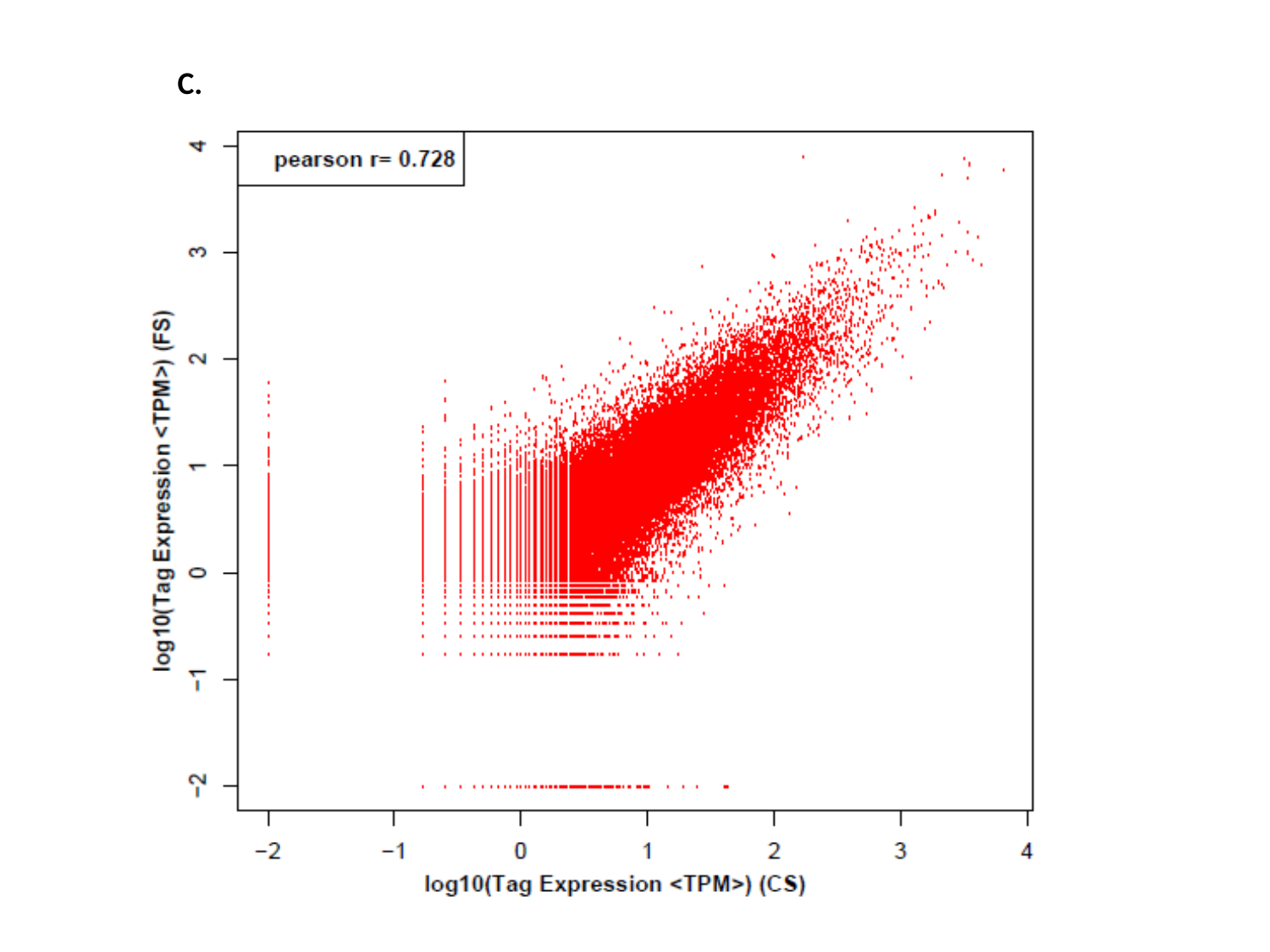

C.

Supplement: S3 Fig — (A) Control vs Cold Stress (CON vs CS), (B) Control vs Freeze Stress (CON vs FS), (C) Cold Stress vs Freeze Stress (CS vs FS). (PPT) [file pone.0121982.s003.ppt]
