# Supplementary material for: DeepSAGE Based Differential Gene Expression Analysis under Cold and Freeze Stress in Seabuckthorn (Hippophae rhamnoides L.)
Source: PLoS One. 2015 Mar 24;10(3):e0121982. doi: 10.1371/journal.pone.0121982 (PMC4372589; doi:10.1371/journal.pone.0121982)
Supplement: S1 Table — (DOC) [file pone.0121982.s006.doc]

| **Gene** | **Annotaion** | **Forward primer (5’-3’)** | **Reverse primer (5’-3’)** | **Product Size (bp)** |
| --- | --- | --- | --- | --- |
| *MAP2K* | Mitogen-activated protein kinase kinase | GTGTGCCTTCTCGGAATGAT | ACCGGTCCAGTGATCCATAA | 147 |
| *ADC* | Arginine decarboxylase | GCTTATGAGGAGGCACTTGG | CATGGCTCGAGTAACTGCAA | 108 |
| *PP2c* | Protein phosphatase 2c | CCTTCGCAGCTTTCAACTTC | CTCATGGCTGATGTTGTTGG | 122 |
| *AP2 /ERF* | AP2/ERF domain-containing transcription factor | GTTGTTTCAGGGCAGAGAGC | CAGCTTCATCTTCACGACCTC | 121 |
| *RPLP0* | 60s acidic ribosomal protein p0 | GGGATGTTGAGAACCTGGAAG | CTGCCCGTGTTGGTTTAGTT | 102 |
| *ATP19a* | Peroxidase ATP19a | AAACATCCCACCTCCAACTG | ATTGTGTGAGCCCCAGAAAG | 105 |
| *SAUR* | SAUR family protein | GGGGGTCTGACAATTCCTTA | AACCCCACACATCTTCCTTC | 167 |
| *HSP70* | Heat shock protein 70 | GACAACAACCAACTCGCAGA | CCTGCACCCTGGTACATCTT | 101 |
| *WD40* | WD-40 repeat family protein | AACCGTGAAGCTATGGGATG | TGCCTTCATTCAGTGACCAG | 157 |
| *KCS* | Beta-ketoacyl- synthase | GGGAAACGACCACTCTATGC | GAGACACCCCAACTTTTCCA | 189 |
| *PAP* | Plastid-lipid-associated protein | TGGGGCTTCGTACTTCAAAC | ATTGTTGTCAAGGGGCACTC | 157 |
| *RNA-h* | RNA helicase | AAGCAACGGCAGACTCTTCT | AATGTGTCTCCACGCTACCC | 127 |
| *CALM* | Calmodulin-related protein | GCTCAGTGATTGTCGCAAGA | AGTTTCAAGCGCGAGACATC | 101 |
| *SIP2* | Galactinol--sucrose galactosyltransferase 6 | GCGTCTTTTTGGGTGAGTTC | AACATAGACAGGGCCACCAC | 113 |
| *CSP1* | Cold shock protein-1 | CGACCGGCTATAGGAGTTGA | ATCACAGGTGGAGGATACGG | 155 |
| *ABC* | ATP binding | ATCTCTGCCTCTTTCGGTGA | AGGCAGAGGTAGGGGATGAT | 124 |
| *MYO* | Myosin | GTGTCTTCAAGCACCCCAAT | GGAGGATGCTGCTTCTCAAG | 138 |
| *SIZ1* | e3 sumo-protein ligase siz1 | GGGCTACGGATTTAGCATCA | CAACCAATGTGCTGCCATAC | 187 |
| *KCS1* | 3-ketoacyl- synthase I | GGACCCAGAGTCGTGATGTT | CGAACCCATAAAGGAGCTGA | 146 |
| *LIP* | Low temprature induced-like protein | AGCCAACCCAACAAAGTCAG | GCAGGCCATGGAAGTTTAAG | 146 |
| *HVA 22* | HVA 22 E protein | CAGTGGCTTGCCTATTGGAT | GAACCAACCAAGCCACAAAC | 124 |
| *SERAT* | Serine acetyltransferase | CGGAGGAGTGGGATGAGATA | CCACCTCCCTCACTATCCAA | 161 |

**Table S1.**  List of genes and primers used in qRT-PCR expression analysis.
